# Medullary bone in an Early Cretaceous enantiornithine bird and discussion regarding its identification in fossils

**DOI:** 10.1038/s41467-018-07621-z

**Published:** 2018-12-05

**Authors:** Jingmai O’Connor, Gregory M. Erickson, Mark Norell, Alida M. Bailleul, Han Hu, Zhonghe Zhou

**Affiliations:** 1 Key Laboratory of Vertebrate Evolution and Human Origins of Chinese Academy of Sciences, Institute of Vertebrate Paleontology and Paleoanthropology, Chinese Academy of Sciences, 100044 Beijing, China; 2 CAS Center for Excellence in Life and Paleoenvironment, 10010 Beijing, China; 3 Department of Biological Science, Florida State University, Tallahassee, FL 32306 USA; 4 Division of Paleontology, American Museum of Natural History, New York, NY 10024-5198 USA; 5 Zoology, University of New England, Armidale, NSW 2351 Australia

## Abstract

Medullary bone is an ephemeral type of bone tissue, today found only in sexually mature female birds, that provides a calcium reservoir for eggshell formation. The presence of medullary bone-like tissues in extant birds, pterosaurs, and dinosaurs distantly related to birds shows that caution must be exercised before concluding that fossils bear medullary bone. Here we describe a new specimen of pengornithid enantiornithine from the Lower Cretaceous Jiufotang Formation. Consisting of an isolated left hindlimb, the three-dimensional preservation contrasts with the crushed preservation characteristic of most Jehol specimens. Histological examinations suggest this resulted from the presence of a thick layer of highly vascular bone spanning the medullary cavities of the femur and tibiotarsus, consistent with expectations for medullary bone in extant birds. Micro-computed tomographic scans reveal small amounts of the same tissue extending into the pedal phalanges. We consider the tissue to be homologous to the medullary bone of Neornithines.

## Abstract (web summary)

Medullary bone is used by modern female birds as a calcium reservoir for eggshell production. Here, O’Connor and colleagues propose criteria for identifying medullary bone in fossils and report medullary bone from a Cretaceous enantiornithine bird fossil.

## Introduction

Medullary bone (MB) is an ephemeral bone tissue known only in extant female birds (Neornithes) that provides a rapidly mobilized calcium reservoir for eggshell production and is metabolized in response to elevated levels of estrogen^1–5^. The name is a misnomer, for although the tissue is most conspicuous in the medullary cavities of avian long bones, it also forms in cancellous spaces throughout the appendicular and axial skeleton^2^. Non-avian MB was first suggested to be present in non-avian dinosaurs (hereafter dinosaurs) based on unusual osseous tissues found in the femur of *Tyrannosaurus rex* MOR (Museum of the Rockies, Bozeman, MT, USA) 1125^6^. In addition to further supporting the ancestral link between birds and theropod dinosaurs^7,8^, this discovery implied this “avian” feature arose surprisingly deep in the dinosaurian tree. Since this discovery, MB (or tissues resembling it, see below) has been described in other dinosaurs, some of which are more distantly related to birds including: two ornithopods—*Dysalotosaurus* and *Tenontosaurus*^9,10^; two non-avian theropods—*Allosaurus*^9^ and an additional *Tyrannosaurus* specimen^11^; and two sauropodomorphs—*Mussaurus*^12^ and a saltasaurine titanosaur^13^. Tissues resembling MB have also been observed in two non-dinosaurian ornithodirans, *Pterodaustro*^14^ and *Bakonydraco*^15^.

Definitive identification of MB in fossil taxa is problematic. Osseous tissues that can be misconstrued for MB (medullary bone-like tissues; MBL) are commonplace in the bones of tetrapods, particularly in the hollow long bones of ornithodirans. Tissues with the potential to appear like MB include: endosteal bone lining medullar cavities^16^; mechanically supporting trabeculae^17^; and trabecular remnants of former metaphyses moved into the diaphyses during ontogeny^16^. In addition, Chinsamy and Tumarkin-Deratzian^18^ showed that tissues remarkably similar to MB occur in some pathological avian bones. Finally, Prondvai and Stein^15^, Chinsamy and colleagues^13^, and Prondvai^19^ have reported MBL with unknown functionality in non-pathological and presumably non-reproductive (rapidly growing, juvenile) dinosaurs and pterosaurs. These issues show that caution should be exercised before concluding that MB reflecting sex and reproductive status is truly present in fossils^15,18–20^. Because of such concerns Schweitzer and colleagues^21^ tried to provide additional support for the presence of MB in *Tyrannosaurus rex* MOR 1125 by means of histochemical analyses. They found that MB in extant ovipositing birds shows significantly greater amounts of glycosaminoglycans, and specifically keratan sulfate, than in the cortical bone (CB). MOR 1125 showed a similar pattern. Nevertheless, subsequent preliminary work by Canoville and colleagues^22^ suggests that pathological bone forming in medullary cavities can also show elevated levels of keratan sulphate. Further testing is required before chemical signatures can serve as a viable means to test for MB in fossils.

The identification of MB in non-avian fossil archosaurs as a means to determine sex and reproductive activity was based on three histological characteristics: (1) location in the medullary cavity; (2) an endosteal origin, marked by a resorption line between the cortex and the hypothetical MB; and (3) a woven arrangement of the collagen fibers, suggesting rapid bone deposition. Given the factors noted above that can produce MBL, these criteria in and of themselves cannot differentiate MB from MBL^18,19^. Hence, some past identifications of MB may be incorrect^12,20^. More precisely, MB is always endosteal in origin (Table 1; Criterion 2), but as previously mentioned, it also forms in cancellous spaces of bones without medullary cavities sensu stricto (e.g., in flat bones of the skull, irregular bones like vertebrae) (Criterion 1). Furthermore, recent studies indicate that MB may form more slowly in some large ratites (possibly due to their large size), which has been interpreted as both lamellar^6,22^ and parallel fibered (Criterion 3)^19^.

**Table 1:** Previous characters used to identify medullary bone (MB) fail to distinguish this female-specific tissue from other medullary bone like tissues (MBL)

Character	Description
1	Occur in the medullary cavity and other cancellous spaces throughout the appendicular and axial skeletons
2	Be of endosteal origin
3	Primarily have a woven arrangement of the collagen fibers indicative of rapid formation (may also be partially parallel-fibered or lamellar in some instances)
4	Co-occur with a periosteal surface free from pathological indicators
5	Line a majority of the medullary cavity (including trabecular surfaces)
6	Be clearly demarcated from the cortical tissue without a graded transition
7	Occur in multiple elements including the tibiotarsus
8	Coincide with reduced growth rates indicative of sexual maturity
9	Often have vascular canals with a doublet or triplet pattern within osteon-like structures (“vascular sinuses”)
10	Have a histochemistry comparable to that of extant avian medullary bone (e.g., shows higher amounts of glycosaminoglycans than that of cortical bone) (unique histochemical signature, yet to be determined)
11	Have a mineral to collagen ratio significantly greater than that of cortical bone

To be strongly considered MB, the bone tissue must fulfill these criteria (characters 10 and 11 cannot be utilized at this time but may prove useful in the future with better understanding of bone histochemistry and the development of new protocols)

MB varies in neornithines, ranging in thickness, skeletal distribution, and orientation of collagen fibers^6,21^. We propose that through a greater number of criteria (Table 1) researchers can better identify probable MB in fossils. In addition to the three previously mentioned criteria, MB should have the following additional six characteristics (Table 1): (4) the periosteal surfaces should be free from indication of pathology [unlike in *Allosaurus* UUVP (Utah Museum of Natural History, Salt Lake City, UT, US.) 5300 in which the periosteum bears a callus]^18^; (5) the MB should line the majority of the medullary cavity [unlike the condition in *Dysalotosaurus* SMNS (Staatliches Museum für Naturkunde, Stuttgart, Germany) T3];^10^ (6) the MB should be clearly demarcated from the CB without a graded transition allowing the MB to often show separation from the cortex during diagenesis;^2,6^ (7) the MB should occur in multiple skeletal elements [in neornithines MB is most commonly seen in the long bones of the hindlimb^23^, most prevalently expressed in the femur^1^, and is universally present in the tibiotarsus^22^. However, in neornithines MB can form extensively throughout the entire skeleton, reportedly occurring in the skull bones and pedal phalanges of some taxa^2,24^; (8) MB should coincide with a reduced growth rate associated with the onset of reproduction making it unlikely to occur in young, rapidly growing individuals [MB in juvenile *Tyrannosaurus* BMRP (Burpee Museum of Natural History, Rockford, Illinois, USA) 2006.4.4 is likely pathological^11^; and finally (9) MB may have vascular sinuses arranged in doublet, triplet or greater patterns (this last criterion may further characterize MB but does not appear to be universally present^6^).

**Fig. 1:**
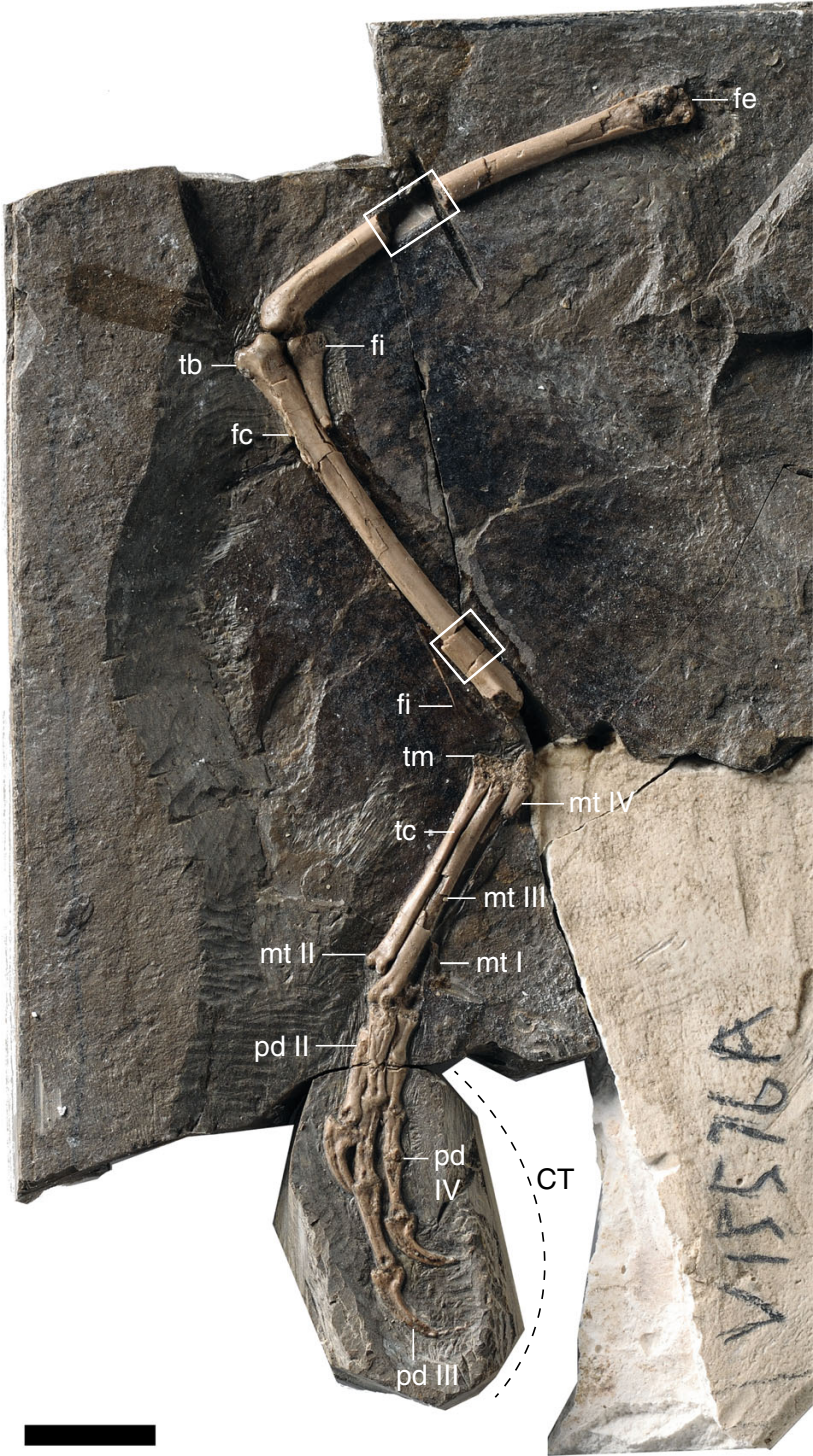
Photograph of the main slab of Pengornithidae indet. (Aves: Enantiornithes) IVPP V15576A represented by a left hindlimb in lateral view. Scale bar equals 10 mm. Dashed line indicates portion of slab removed for CT scanning. Anatomical abbreviations: fc, fibular crest; fe, femur; fi, fibula; mt, metatarsal; pd, pedal digit; tc, area of attachment of the m. tibialis cranialis; tm, tarsometatarsus

Here we describe a new specimen of pengornithid enantiornithine bird from the Early Cretaceous Jiufotang Formation of China, which records the youngest stage of the Lower Cretaceous Jehol Biota (Fig. 1). Using gross morphology, histologic microstructure, tissue distributions, comparison with neornithines and purported non-neornithine fossil MB, and the nine aforementioned neornithine MB correlates, we demonstrate that the new specimen bears what is best interpreted as MB in its long bones. This represents the most compelling evidence for MB in a Mesozoic bird at this time, strongly suggesting this tissue evolved outside the crown lineage.

## Results

### Identification

The new specimen IVPP (Institute of Vertebrate Paleontology and Paleoanthroplogy, Chinese Academy of Sciences, Beijing, China) V15576 is an isolated, articulated left hindlimb preserved in three dimensions. This stands in contrast to the typically crushed preservation of most other Jehol specimens (Fig. 1). Traces of crural feathers are weakly preserved. The fossil was collected by an IVPP team in the Lower Cretaceous Jiufotang Formation^25^ near Chifeng, Inner Mongolia Autonomous Region, China. The presence of a femur nearly as long as the tibiotarsus, elongate fibula subequal in length to the tibia, presence of metatarsal V, and a proportionately elongate metatarsal I (38% length of metatarsal III; 33–42% in pengornithids) and hallux clearly allow referral of IVPP V15576 to the Pengornithidae, a diverse clade of basal enantiornithines^26^. This is the first pengornithid collected from Inner Mongolia, but without additional material for the specimen, it cannot be identified to the generic level. IVPP V15576 (femur length, 38.8 mm; incomplete tibiotarsus, 37 mm; metatarsal III, 21.9 mm; metatarsal I, 8.4 mm) is similar in size to *Parapengornis* IVPP V18687 and pengornithid IVPP V16832 but much smaller than *Pengornis houi* IVPP V15536 (femur length, 48 mm)^27,28^. Unfortunately, the distal end of the tibiotarsus is not preserved and the proximal end of the tarsometatarsus is abraded, making it difficult to assess the degree of fusion among these compound elements. It appears fusion of the proximal tarsometatarsus may have been incomplete.

### Femoral histology

**Fig. 2:**
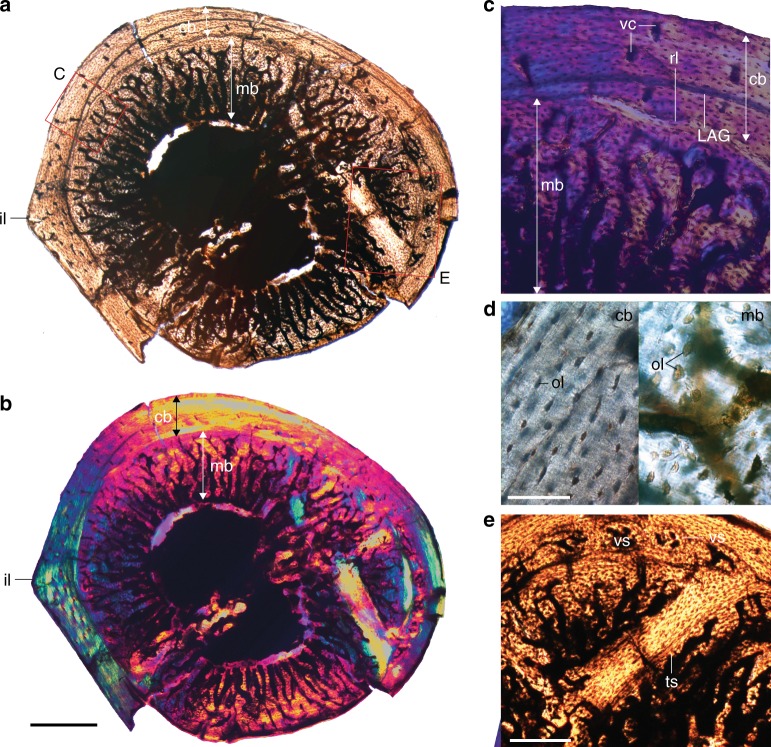
Osteohistology of the femur of pengornithid IVPP V15576. **a** Full cross-section under normal light; **b** cross-section under polarized light; **c** LAG in femur approached by endosteal resorption; **d** close up of the osteocyte lacunae in the cortical bone compared to the larger and more irregular osteocyte lacunae in the medullary bone; **e** close up of region preserving vascular sinuses and a structure interpreted as a trabecula. Anatomical abbreviations: cb, cortical bone; il, intermuscular line; LAG, line of arrested growth; mb, medullary bone; ol, osteocyte lacunae; rl, resorption line; vc, vascular canal; vs, vascular sinus; ts, trabecular strut. Scale bar for **a** and **b** is 500 µm; scale bar for **d** is 50 µm; scale bar for **e** is 200 µm

The transverse diaphyseal thin-section from the femur of IVPP V15576 reveals an external layer of CB ~190–290 µm in width, and a thicker more-vascularized layer (hereafter referred to as MB) between 440 and 890 µm in width that spans circumferentially around most of the medullar cavity (Fig. 2). The CB consists of poorly vascularized parallel-fibered bone with mostly simple, longitudinal vascular canals together with a few reticular canals. CB osteocyte lacunae are more organized and smaller than those found in the MB (Fig. 2d). The cortex is interrupted by a line of arrested growth (LAG) (Fig. 2c). No outer circumferential layer (OCL), nor inner circumferential layer (ICL) is present, suggesting that the bird had not reached skeletal maturity^29,30^. Near the caudal intermuscular line (a bony ridge extending distally from the caudal surface to the lateral surface that marks the boundary between the m. femorotibialis externus and femoral adductors)^31,32^ the LAG reaches the contact between the CB and MB (i.e., the LAG is obliterated by the resorption line). Opposite the intermuscular line the LAG approaches the periosteal surface. This presumably reflects extensive ontogenetic osseous drift^33^. The periosteal surface of the bone is smooth and lacks any indication of pathology^18^.

**Fig. 3:**
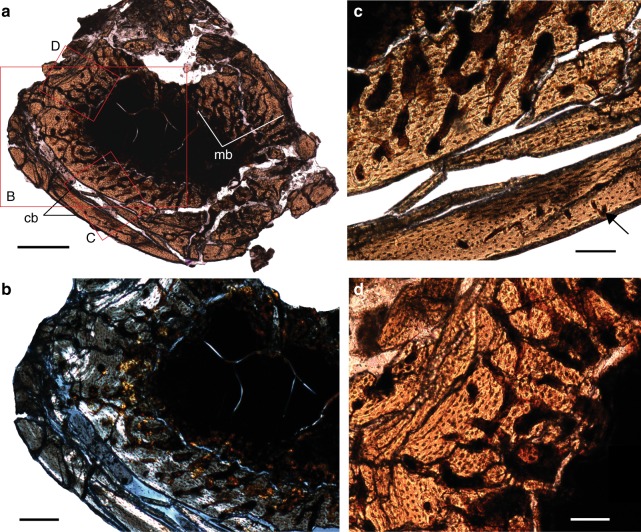
Osteohistology of the tibiotarsus of pengornithid IVPP V15576. **a** Full cross-section; **b** close up under polarized light; **c** close up of the longitudinal canal near the periosteal surface (black arrow) indicating active growth; **d** close up showing radial vascular canals and spicular trabeculae of the MB. Scale bar in **a** equals 500 µm; scale bar in **b** equals 200 µm; scale bars in **c** and **d** equal 100 µm

The contact between the CB and MB is uneven, with the characteristic scalloped resorption line created by osteoclasts^34^ prior to the deposition of MB in extant birds. The MB consists of extremely vascularized woven to parallel-fibered bone, with numerous radial vascular canals, and spicular trabeculae radiating inward towards the center of the medullary cavity. These are oriented approximately perpendicular to the periosteal surface. The vascular canals get wider as they approach the center of the medullary cavity. Opposite to the intermuscular line, a small area of MB located near the CB possesses a few canals organized into small groups within osteon-like structures (Fig. 2e). Some of those groups show a reticular pattern, while others are simply groups of longitudinal canals (Fig. 2e). The latter look extremely similar to the doublet and triplet vascular patterns, referred to as “vascular sinuses” in the MB of *Tyrannosaurus rex* and extant ratites (Fig. 3g and S3 in Schweitzer et al. ^6^). However, one of these triplet vascular sinuses appears to occur in the CB. Nearing these “vascular sinuses”, within the medullary cavity, a thick strut of avascular parallel-fibered bone is also lined with MB. Like the CB, the bone is birefringent and the osteocyte lacunae have a more organized appearance suggesting this may represent a trabecular strut. MB also develops on the naturally-occurring metaphyseal bone trabeculae of living birds^21^. Although not directly connected to the CB in our cross section, it may be that the trabecula extends into the medullary cavity at an angle passing through our sectioned plane (Fig. 2).

The contact between CB and MB is clearly visible under polarized light, with the MB being only weakly birefringent (Fig. 2b). This is consistent with relatively rapid deposition and a lower amount of collagen fibrils^35^. Rapid bone deposition is also suggested by the larger size and bulbous appearance of the osteocytes relative to those in the CB. The low birefringence suggests the MB may have formed more slowly than in most extant taxa. In one area the MB is preserved but not the CB, indicating weakness at the basal layer as in extant avian MB^6^. Notably, the MB is more than double the thickness of the CB in most of the femur. This is consistent with the peak condition in many modern birds during the last twelve hours before oviposition^36^.

### Tibiotarsal histology

The tibiotarsus suffered more diagenetic alteration than the femur. It is heavily crushed and the damage slightly obscures detailed observations that were feasible in the femur (Fig. 3a). Its cortex is thinner than that of the femur (180–190 µm), with a larger medullary cavity and a proportionately thinner layer of MB (360–580 µm). This condition is also observed in living birds^1^. The CB consists of parallel-fibered bone, with simple, longitudinal canals located close to the periosteal surface. No LAG, OCL, nor ICL can be confidently identified. As in the femur, the MB consists of both woven and parallel-fibered bone. Osteocyte lacunae also are generally smaller in the tibiotarsus when compared to those in the femur. In some places the MB and CB are delaminated (Fig. 3).

### Computed tomographic (CT) scans of pedal phalanges

**Fig. 4:**
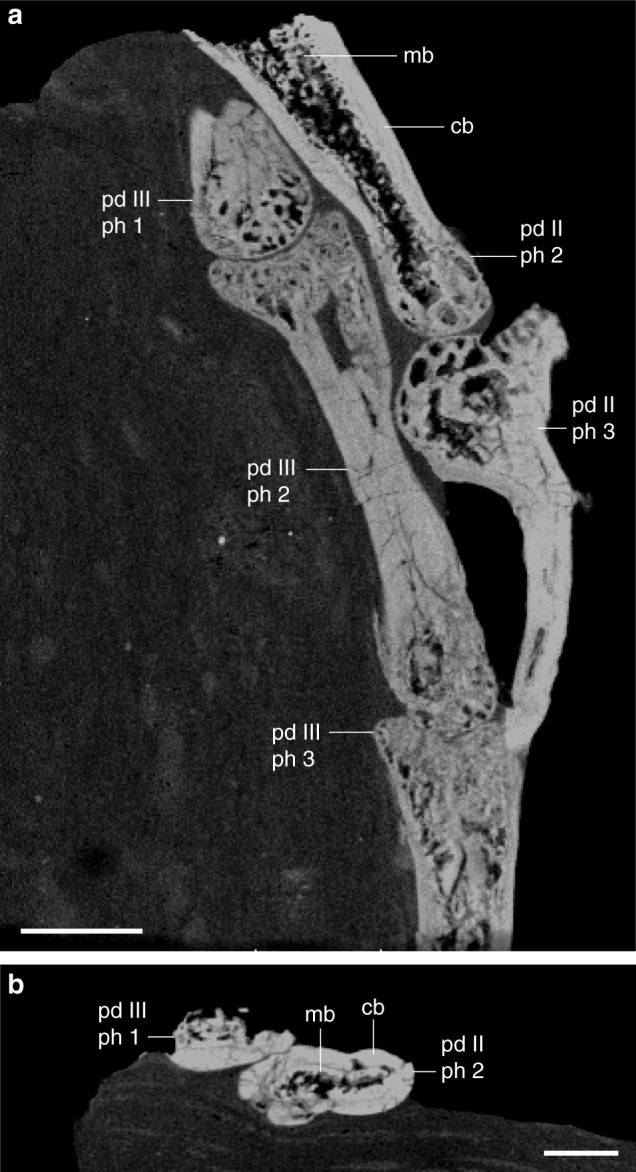
CT-scans of the distal pedal phalanges showing the penultimate phalanx of pedal digit II with small amounts of medullary bone lining the internal cavity. **a** Medial slice along the *x*-axis showing pedal digits II and III; **b** same section, *y*-axis. Scale bar equals 1000 μm. Anatomical abbreviations not listed in Fig. 2 caption: ph, phalanx

A crack allowed for a portion of the slab containing the distal pedal phalanges to be removed (Fig. 1) and µCT scanned (Fig. 4). Although these scans do not provide a detailed histological examination (e.g., no osteocyte lacunae, no collagen fiber orientation), they do show some useful microarchitectural details. Notably, they reveal small amounts of MB in the non-ungual pedal phalanges. This tissue is easily identified by its cancellous architecture within the medullary cavity in longitudinal view (Fig. 4a). In cross-section a faint line can be seen demarcating the internal MB and the external CB (Fig. 4b).

### Comparison with neornithines

IVPP V15576 preserves unusual bone tissue in the medullary cavities (Criterion 1) of the hindlimb bones. As in the MB of living birds, the tissue is clearly endosteal in origin as indicated by the presence of a resorption tideline (Criterion 2). The MB in IVPP V15576 is weakly birefringent compared to the CB (Fig. 2b), consistent with relatively rapid deposition (Criterion 3)^35^. The tissue is primarily woven but also somewhat parallel-fibered in both the femur and tibiotarsus (Criterion 3). This is consistent with observations of some paleognath neornithines^21^. The periosteal surfaces of all the preserved elements in IVPP V15576 are smooth, without indication of pathology (Criterion 4). As in most living birds, the cancellous bone tissue in IVPP V15576 is found lining nearly the entire medullary cavity (Criterion 5) and has a clearly visible limit with the CB, often leading to delamination in fossils (Criterion 6). In one area the MB is preserved but not the CB, indicating weakness at the basal layer as in extant avian MB (Criterion 6)^6^. The MB is found throughout the preserved elements indicative of a systemic process. Where preservation permits comparison (only hindlimb), IVPP 15576 matches living birds in extent and distribution (Criterion 7), being most strongly developed in the femur (Fig. 2)^1^. The tissue apparently extends into the non-ungual pedal phalanges (Fig. 4), where it is known to occur in some extant neornithine taxa^2^. The CB of the femur is poorly vascularized and interrupted by a LAG indicating a period of reduced growth rate. This strongly suggests that IVPP V15576 was not a rapidly growing subadult (Criterion 8). Vascular sinuses arranged in triplet and doublet patterns like those reported in the MB of some extant birds and *T. rex* (MOR 1125)^6^ are also present in the femoral section of IVPP V15576 (Fig. 2e) (Criterion 8; Tables 1, 2). In IVPP V15576 MB is found on the trabecular strut, consistent with the naturally occurring metaphyseal bone trabeculae in living birds^21^. Comparison of the purported MB in IVPP V15576 with living, reproductively active female birds reveals no observable differences. Thus we conclude that this fossil bird preserves MB homologous to that in female neornithines.

**Table 2:** All previously described occurrences of purported MB in Mesozoic fossils scored across the nine features (Table 1) that, together, should allow identification of preserved tissue as MB (present indicated by x, absence with a dash)

Taxon	Collection #	Clade	Element(s)	1	2	3	4	5	6	7	8	9	10	11	Identification	Revised	Reference
*Confuciusornis*	DNHM D1874	Aves	Humerus	x	?	?	x	–	?	–	x	–	?	?	MB	*Unlikely*	^37^
Pengornithid enantiornithine	IVPP V15576	Aves	Femur, tibiotarsus	x	x	x	x	x	x	x	x	x	?	?	MB	–	This study
Transylvanian dinosaur	BM R 5505	Dinosauria	long bone	x	x	x	–	?	x	?	?	?	?	?	Osteopetrosis		^18^
*Tenontosaurus*	OMNH 34784	Ornithopoda	Femur, tibia	x	x	?	x	?	x	x	?	?	?	?	MB	*Unlikely*	^9^
*Dysalotosaurus*	SMNS T3	Ornithopoda	Tibia	x	x	x	x	–	x	?	x	?	?	?	MB	*Unlikely*	^10^
*Dysalotosaurus*	GPIT/RE/5109	Ornithopoda	Fibula	x	x	x	x	–	x	?	x	?	?	?	MB?	*Unlikely*	^10^
*Mussaurus*	MLP 61-III-20-22	Sauropoda	Femur	x	x	–	x	–	x	?	x	?	?	?	MB	Pathological	^12, 20^
*Saltasaurus*	PVL 4017–140	Sauropoda	caudal vertebra	x	x	x/–	x	–	–	?	?	?	?	?	Not MB		^13^
*Saltasaurus*	PVL 4017–113	Sauropoda	osteoderm	–	x	x	x	–	–	?	?	?	?	?	Not MB		^13^
*Saltasaurus*	PVL 4017– 127	Sauropoda	metatarsal	x	x	x	–	x	x	?	?	?	?	?	Pathology		^13^
Wealden Sauropod		Sauropoda	vertebra	x	x	x	?	?	–	?	?	?	?	?	Pathology		^48^
*Allosaurus*	UUVP 5300	Theropoda	Tibia	x	x	x	–	?	x	?	?	?	?	?	MB	Osteopetrosis	^9, 18^
*Tyrannosaurus rex*	MOR 1125	Theropoda	Femur	x	x	x	x	?	x	?	x	x	?	?	MB		^6, 21^
*Tyrannosaurus rex*	BMRP 2006.4.4	Theropoda	Tibia	x	?	?	?	?	?	–	–	?	?	?	Pathology		^11^
*Bakonydraco*	MTM V PAL 2007.111.1	Pterosauria	mandibular symphysis	x	x	x	x	–	x	–	–	–	?	?	Not MB	Pathological?	^13, 15^
*Pterodaustro*	MHIN-UNSL-GEO V 382	Pterosauria	Femur	x	x	x	x	?	x	?	x	?	?	?	MB?	*Unlikely*	^14^

Published data often did not allow for some characters to be scored. Characters 10 and 11 cannot be utilized at this time but may prove useful in the future with better understanding of bone histochemistry and the development of new protocols. The column 'identification' refers to the original identification; the 'revised' column presents alternative interpretations published after the initial identification with those in italics representing new views presented in this paper

## Discussion

IVPP V15576 preserves what we interpret as extensive MB throughout the femur and tibiotarsus (Figs. 2,3) that likely extended into the non-ungual pedal phalanges (Fig. 4). This represents the only demonstrated occurrence of MB in the Enantiornithes, the dominant clade of terrestrial birds during the Cretaceous. Furthermore, according to our proposed criteria for identifying MB in fossils, IVPP V15576 preserves the most credible evidence of this tissue’s presence in a non-neornithine archosaur thus far (Table 2; see Supplementary Note 1).

Among Mesozoic birds, MB has been previously reported only in the basal pygostylian, *Confuciusornis* (Aves: Pygostylia) DNHM (Dalian Natural History Museum, Dalian, China) D1874^37^. Although the humerus, ulna, and tibiotarsus were all sampled, MB was only found in the humerus and to a lesser extent in the ulna. This contrasts with the distribution in extant ovipositing birds where all studied taxa show the presence of MB in the tibiotarsus^22^. Differences in bone texture (i.e., cancellous versus compact) are cited in support of the interpretation of small bone fragments in the medullary cavity of the humerus as representing MB^37^. However, the alleged MB is very limited in distribution and associated with fragments that are most likely from the cortex. Thus, based on our more stringent criteria, evidence for MB in this specimen is equivocal at best (Table 2). However, if this identification is correct, it is notable that the MB is most developed in the humerus and not at all present in the tibiotarsus. This is in stark contrast to MB in all studied extant neornithines^22^. The pattern in *Confuciusornis* is opposite to that observed in most living birds, in which the pneumatic humeri contain very little MB and the femora and tibiotarsi have the most^2^. If in fact the tissue is MB, the findings may be the result of the fact the humerus was probably apneumatic in basal birds^38^, suggesting some early birds distributed MB in the skeleton differently from extant neornithine taxa.

If conclusions about the presence of MB in the pengornithid IVPP V15576 are correct, this specimen represents a reproductively active female. The presence of vascular canals close to the periosteum and the absence of an OCL indicate IVPP V15576 was still actively growing at the time of death, confirming inferences that enantiornithines, like non-avian dinosaurs^9,39^ reached reproductive maturity (RM) before somatic maturity (SM). This is unlike the condition observed in most neornithines, in which RM is coincident with SM^29,40^ (although some neornithines have secondarily evolved protracted growth)^41^. Assuming this represents the primitive condition at the base of Aves, this suggests that the modern condition seen in living birds (i.e., RM after SM) is limited to a subset of the Ornithuromorpha. The LAG present in the femur of IVPP V15576 may represent the acquisition of RM, as suggested in a previously described enantiornithine preserving similar histological features^9,29^. The CB of IVPP V15576 shows vascularization after the inferred acquisition of RM, whereas the CB becomes avascular after the deposition of the double-LAG in the femur of enantiornithine STM (Shandong Tianyu Museum of Nature, Pingyi, China) 29-8^29^. This adds to the mounting body of evidence that enantiornithines were diverse in their growth strategies^42^. This is true even within the Pengornithidae; although nearly the same size, the slightly larger *Parapengornis* IVPP V18687 (femur length, 39.8 mm) preserves very different femoral histology with much greater vascularization and no LAGs or ICL. These are features consistent with a younger, more rapidly growing individual^27^.

Two Early Cretaceous birds preserving ovarian follicles were not found to show MB^29^. There are three possible interpretations for this absence: the circular structures preserved in the abdominal cavity of *Jeholornis* STM2-51 and enantiornithine STM29-8 in fact do not represent ovarian follicles^43^; taphonomy resulted in the loss of this delicate feature; or MB was not yet present at the time of death in these specimens. The latter implies the follicles were not as mature as previously inferred^29,44^. Rapid yolk deposition is inferred to be absent in enantiornithines and thus folliculogenesis most likely occurred over an extended period, as in crocodylians^45^. This could explain the relatively large number of specimens found preserving follicles (*n* = 10)^45,46^. In pigeons, MB only appears a few days before ovulation^1^ and in some taxa MB is only present for a matter of hours^23^. Given the ephemeral nature of avian MB, its absence in the two sampled specimens preserving developing follicles is not surprising. However, in enantiornithines the presence of MB may have persisted for slightly longer intervals than in neornithines. Consistent with the slower growth rates observed in enantiornithines^29,30,47^ and perhaps an extended period of reproductive activity, the MB tissue in IVPP V15576 appears to be less woven than in most neornithines, also including some parallel-fibered tissue. This suggests enantiornithine MB, like enantiornithine CB, may have formed at a slower rate than that of most similarly sized modern birds.

Because pathological endosteal tissues resembling MB can form in extant birds and MBL occurs in fossils lacking identifiable evidence for both reproductive activity and pathology, unequivocal identification of MB requires more than morphological similarity (Tables 1, 2). Work has already begun to identify a unique histochemical signature able to identify MB in well preserved fossils (Criterion 10)^21^. However, further analysis is required before it will be possible to definitively identify MB through such methods^22^. Therefore, at this time, no published non-neornithine specimen preserving MB can be unequivocally identified as such. MB is also characterized by (Criterion 11) a lower content of collagen fibrils and a higher degree of calcification^1^ and it may be possible in the future to develop techniques allowing the determination of these characteristics in the MB of IVPP V15576 using SEM, microradiography, microCT, and/or other methodologies. In summation, pending potential future histochemical analyses and the development of new techniques, we suggest the cancellous MB tissue present in the medullary cavity of the femur, tibiotarsus, and non-ungual pedal phalanges in IVPP V15576 most likely represents MB based on standard paleohistological examinations.

## Methods

### Histological analysis

The femoral diaphysis sample was extracted using a micro-saw and embedded in a clear epoxy resin (Epoxyset; Allied High Tech Products Inc., Rancho Dominguez, CA, USA). The sample was serially sectioned in the transverse plane in 1.5 mm increments using a slow-speed diamond saw (Isomet 1000; Buehler Inc., Lake Bluff IL, USA). The sections were affixed to petrographic microscope slides using epoxy and rotary-sanded (Rotopol 11; Struers Inc., Cleveland, OH, USA) to ~ 100 μm thickness using descending grades (220–1200 grit) of carbide sandpaper using water as a lubricant then polished using an alumina slurry. The femur was visualized and photographed using both incident light SZX12; Olympus Inc., Tokyo Japan) and polarized petrographic microscopy (BX60; Olympus Inc., Tokyo Japan).

The tibiotarsus sample was taken using a micro-saw, embedded in EXAKT Technovit 7200 one-component resin, and allowed to dry for 24 h. The sample was then cut and polished until the desired optical contrast was reached (slice thickness between 100 and 150 µm). The sample was viewed under normal and polarized light using a Leica DM- RX polarizing microscope. It was photographed using a Zeiss AxioCam MRc5 and using the Axiovision SE66 4.9 software. Measurements were taken with the computer software Fiji (ImageJ) v.1.0.

### CT-scans

The specimen was scanned using the 225-kV micro-CT at the Key Laboratory of Vertebrate Evolution and Human Origin of CAS, IVPP, CAS, Beijing, China. The part of the slab containing the pedal phalanges was removed from the main block along an existing crack and scanned at a detector resolution of 7.8 μm per pixel, and three-dimensional reconstructions were created with the software Avizo (version 8.1).

## Electronic supplementary material

Supplementary Information

## Data Availability

IVPP V15576 is reposited at the Institute of Vertebrate Paleontology in Beijing. All data are available upon reasonable request.
