# Supplementary material for: Medullary bone in an Early Cretaceous enantiornithine bird and discussion regarding its identification in fossils
Source: Nat Commun. 2018 Dec 5;9:5169. doi: 10.1038/s41467-018-07621-z (PMC6281594; doi:10.1038/s41467-018-07621-z)
Supplement: Supplementary file 1 — Supplementary Information [file 41467_2018_7621_MOESM1_ESM.pdf]

Supplemental Information

Medullary bone in an Early Cretaceous enantiornithine bird and discussion regarding its identification in fossils

O'Connor et al.

Supplementary Note 1: Assessment of medullary bone and medullary bone-like tissues in non-neornithine fossils

Pengornithid IVPP V15576 (Aves: Enantiornithes)

The periosteum of all the preserved elements is entirely healthy, which suggests the MB is not pathological. The extent of the MB throughout the femur, tibiotarsus and even pedal phalanges indicates a systemic process, rather than a pathology which may only affect part of the system. The morphology and distribution of the bone tissue in the medullary cavity is consistent with observations from living birds.

***Confuciusornis* DNHM D1874 (Aves: Pygostylia)**

This specimen[1] does not explicitly reveal tissue that is readily identifiable as medullary bone (where other instances of MBL are clearly visible, the purported tissue in this specimen is not) and the absence of MB in the femur (where it occurs in all reported neornithines, in IVPP V15576, and *Tyrannosaurus* MOR1125) further suggests that the bone fragments considered MB were misidentified.

Transylvanian dinosaur BM R 5505 (Dinosauria)

MBL originally identified as osteopetrosis, based on the presence of an unhealthy periosteum[2].

***Tenontosaurus* OMNH 34784 (Ornithopoda)**

The MBL (or MB) figured by Lee and Werning[3] differs morphologically from neornithine MB. Although limited by the published figures, it looks like the MBL of *Tenontosaurus* presents a different mineralization process than neornithine MB, and also from its own CB. It is hard to assess. The MBL may be diagenetically altered; if not, it appears to have a granular appearance, reminiscent of mineralization found in ossified tendons. If it is being resorbed, it does not present any resorption line. The *Tenontosaurus* present a 'granular' appearance, reminiscent of mineralized tendons, which presumably do not mineralise like periosteal or endosteal bone. It is almost impossible to assess, but it clearly does not resemble standard medullary bone

***Dysalotosaurus* SMNS T3 (Ornithopoda)**

The purported MB only lines a small portion of the medullary cavity in cross-section and also occurs lining resorption cavities within the cortical bone[4]. This distribution is not consistent with identification as MB based on comparison with Neornithes.

***Dysalotosaurus* GPIT/RE/5109 (Ornithopoda)**

The possible MB differs morphologically from neornithine MB, but because the images are so small it is impossible to assess further.

***Mussaurus* MLP 61-III-20-22 (Sauropoda)**

Although originally identified as potential MB[5], the tissue was reinterpreted as pathological, most-likely avian osteopetrosis[6], based on the fact the MBL shows densely packed osteocyte lacunae and a poorly fibrous matrix, features consistent with this disease and distinct from the large globular osteocyte lacunae present in MB.

***Saltasaurus* PVL 4017–140 (Sauropoda)**

Identification in this caudal vertebra is uncertain[7]. However, it is unlikely that sauropods would have required MB given their size and reproductive strategy. Furthermore, the MBL in this element lines only a portion of the medullary cavity and is not clearly demarcated from the cortical bone, both of which suggest the tissue does not represent MB.

***Saltasaurus* PVL 4017–113 (Sauropoda)**

Identification in this osteoderm is uncertain[7]. However, it is unlikely that sauropods would have required MB given their size and reproductive strategy. Being directly ossified, most osteoderms do not have true medullary cavities, although hollow osteoderms are known in large taxa[8] – however, this is not the case in this specimen in which the osteoderm is primarily solid with a few small internal cavities. Furthermore, the unusual tissue in this specimen is restricted to a small area and not distinctly separated from the normal bone tissue, all-together making it unlikely that the unusual tissue in this specimen represents MB.

***Saltasaurus* PVL 4017– 127 (Sauropoda)**

Co-occurs with an unhealthy periosteum suggesting the MBL is pathological in origin[7].

Wealden Sauropod (Sauropoda)

Unlike MB, the MBL in this specimen is not clearly demarcated from the cortical bone and is regarded as pathological[9].

***Allosaurus* UUVP 5300 (Theropoda)**

The MBL figured by Lee and Werning[3] differs morphologically from neornithine MB and the periosteum preserves indicators of pathology, leading others to re-identify the MBL as pathological due to avian osteopetrosis[2].

***Tyrannosaurus rex* MOR 1125 (Theropoda)**

There is no evidence (e.g., unhealthy periosteum) to indicate the preserved tissue is not MB, but given the unusual MBL tissues that have been documented in several other specimens (including another *T. rex*, see below), identification of the preserved tissue as MBL also cannot be ruled out at this time. Unfortunately, the femur is isolated and it cannot be determined if the tissue occurred in multiple elements, as does MB in neornithines.

***Tyrannosaurus rex* BMRP 2006.4.4 (Theropoda)**

The MBL in this specimen was originally identified as pathological, based on the fact that this specimen represents a young, rapidly growing individual[10].

***Bakonydraco* MTM V PAL 2007.111.1 (Pterosauria)**

Given that pterosaurs have soft eggshells, it is unlikely they would have required a calcium reservoir during egg-laying. The tissue described in specimens of *Bakonydraco* was never referred to as MB homologous to that of Neornithes. The MBL in *Bakonydraco*[11] is found in a number of individuals but is found only in the mandibular symphysis, not occurring in any of the bones that more typically display MB in Neornithes. In addition, the MBL occurs in some young, rapidly growing individuals. These two indicators strongly suggest that it is not MB. Although initially ruled out, this MBL may potentially represent some pathology that infected a large portion of the population[7].

***Pterodaustro* MHIN-UNSL-GEO V 382 (Pterosauria)**

Given that pterosaurs have soft eggshells[12], it is unlikely they would have required a calcium reservoir during egg-laying.

Supplemental References

1 Chinsamy, A., Chiappe, L. M., Marugán-Lobón, J., Gao, C.-H. & Zhang, F.-J. Gender identification of the Mesozoic bird *Confuciusornis sanctus*. *Nature Communications* **4**, 1-5, doi:10.1038/ncomms2377 (2013).
2 Chinsamy, A. & Tumarkin-Deratzian, A. Pathologic bone tissues in a turkey vulture and a nonavian dinosaur: implications for interpreting endosteal bone and radial fibrolamellar bone in fossil dinosaurs. *The Anatomical Record* **292**, 1478-1848 (2009).
3 Lee, A. H. & Werning, S. Sexual maturity in growing dinosaurs does not fit reptilian growth models. *Proceedings of the National Academy of Sciences* **105**, 582-587, doi:10.1073/pnas.0708903105 (2008).
4 Hübner, T. R. Bone Histology in *Dysalotosaurus lettowvorbecki* (Ornithischia: Iguanodontia) – Variation, Growth, and Implications. *PLoS ONE* **7**, e29958 (2012).
5 Cerda, I. A. & Pol, D. Evidence for gender-specific reproductive tissue in a basal sauropodomorph dinosaur from the Late Triassic of Argentina. *Ameghiniana* **50**, 11-12R (2013).
6 Cerda, I. A., Chinsamy, A. & Pol, D. Unusual endosteally formed bone tissue in a Patagonian basal sauropodomorph dinosaur. *The Anatomical Record* **297**, 1385-1391 (2014).
7 Chinsamy, A., Cerda, I. A. & Powell, J. E. Vascularised endosteal bone tissue in armoured sauropod dinosaurs. *Scientific Reports* **6**, 1-9 (2016).
8 Rogers, K. C., D'Emic, M., Rogers, R., Vickaryous, M. & Cagan, A. Sauropod dinosaur osteoderms from the Late Cretaceous of Madagascar. *Nature Communications* **2**, 1-5 (2011).
9 Reid, R. E. H. Bone histology of the Cleveland-Lloyd dinosaurs and of dinosaurs in general, Part I: Introduction: Introduction to bone tissues. *Brigham Young Univ Geol Stud* **41**, 25-72 (1996).
10 Tremaine, K., Woodward Ballard, H. & Horner, J. R. in *Society of Vertebrate Paleontology* 240 (Journal of Vertebrate Paleontology, Berlin, 2014).
11 Prondvai, E. & Stein, K. H. W. Medullary bone-like tissue in the mandibular symphyses of a pterosaur suggests non-reproductive significance. *Scientific Reports* **4**, 1-9 (2014).
12 Wang, X.-L. *et al.* Egg accumulation with 3D embryos provides insight into the life history of a pterosaur. *Science* **358**, 1197-1201 (2017).
